# M. Rossi’s Case of Recovery after Extirpation of the Uterus Immediately after Delivery

**Published:** 1842-08-06

**Authors:** 


*Recovery after Extirpation of the Uterus immediately after Delivery.* By M. Rossi. A woman of a feeble constitution had a good delivery; the placenta also came away easily.

The midwife who attended her having pushed her hand into the vagina felt a tumor which she mistook for a second child. She accordingly pulled with such force at this tumor, which happened to be the uterus itself, that she tore it from its attachments, and then, by means of a knife, separated it from the vagina and removed it entire. The woman, however, recovered in about thirty days. M. Rossi, who read the history of the case before the medical section of the Congress at Florence, exhibited the uterus which had been thus removed. He was far, however, from thinking that the same success would attend the removal of the entire uterus in cancerous affections of that organ, as, in addition to the real difficulties and dangers of the operation, it was often difficult to tell how far the neighboring organs were affected, or whether the disease might not return.—*Ibid, from Il Raccoglitore Medico,* October, 1841.

